# Brain Responses to Emotional Stimuli after Eicosapentaenoic Acid and Docosahexaenoic Acid Treatments in Major Depressive Disorder: Toward Personalized Medicine with Anti-Inflammatory Nutraceuticals

**DOI:** 10.3390/jpm10040283

**Published:** 2020-12-16

**Authors:** Cheng-Hao Tu, Chun-Ming Chen, Chuan-Chih Yang, Piotr Gałecki, Kuan-Pin Su

**Affiliations:** 1 Graduate Institute of Acupuncture Science, China Medical University, Taichung 404333, Taiwan; lordowentu@gmail.com; 2 Mind-Body Interface Laboratory, Department of Psychiatry, China Medical University Hospital, Taichung 404332, Taiwan; jinmingc@yahoo.com.hk (C.-M.C.); chuan.chih.yang@gmail.com (C.-C.Y.); 3 Department of Medical Imaging, China Medical University Hospital, Taichung 404332, Taiwan; 4 Department of Adult Psychiatry, Medical University of Lodz, 91-229 Lodz, Poland; piotr.galecki@umed.lodz.pl; 5 Graduate Institute of Biomedical Sciences, College of Medicine, China Medical University, Taichung 404333, Taiwan; 6 An-Nan Hospital, China Medical University, Tainan 709204, Taiwan

**Keywords:** omega-3 polyunsaturated fatty acids (n-3 PUFAs), eicosapentaenoic acid (EPA), docosahexaenoic acid (DHA), major depressive disorder (MDD), functional magnetic resonance imaging (fMRI)

## Abstract

N-3 polyunsaturated fatty acid supplements improve the symptoms of major depressive disorder (MDD) in randomized-controlled trials and meta-analyses, with the higher efficacy from anti-inflammatory eicosapentaenoic acid (EPA) than brain-dominant docosahexaenoic acid (DHA). To investigate the specific brain mechanisms of the anti-inflammatory anti-depressant nutraceutical compounds, we recruited 24 MDD subjects in this double-blind, head-to-head study with a 12-week EPA or DHA treatment (clinical trial registration number: NCT03871088). The depression severity was assessed by Hamilton depression rating scale (HAM-D). Brain responses to emotional stimuli were measured by a 3-Tesla MRI. The correlation between HAM-D scores and brain responses also were tested. Compared to 18 healthy controls, the brain responses of untreated 24 MDD patients mainly revealed hypoactivity in the regions associated with emotion perception and emotion control when processing positive emotion. After treatment, more remitted MDD patients have been observed in the EPA as compared to the DHA groups. In addition, the EPA, but not DHA, treatment revealed increased activity in the regions associated with emotion perception and cognitive control when processing positive emotion. The correlation analysis further revealed negative correlation between HAM-D scores and brain responses in cognitive control regions. The results of this study may imply the compensatory brain responses of cognitive and emotion controls by EPA but not DHA and underpin personalized medicine with anti-inflammatory nutraceuticals toward depression treatments.

## 1. Introduction

Depression is an increasing mental health problem around whole world. It has been estimated that 6.7% adults in U.S. had at least one major depressive episode in 2014 [1]. Patients with major depressive disorder (MDD) have negative affective bias in terms of decreased responsiveness to positive emotion stimuli and/or increased responsiveness to negative emotion stimuli [2,3]. The negative affective bias may be underpinned with impaired affective perception and/or affective cognitive control [4]. The MDD patients exhibit not only selective attention bias but also affective facial perception bias [5,6]. Recent study further reported that the biased affective facial recognition may reflected the clinical state, but not trait, of MDD [7]. Hence, the affective stimuli may be a useful tool to probe the static changes of the MDD.

Omega-3 polyunsaturated fatty acids (ω-3 or n-3 PUFAs), mainly eicosapentaenoic acid (EPA) and docosahexaenoic acid (DHA), are essential nutritional compounds with potential preventive and therapeutic effects against several psychiatric disorders, especially depression [8,9,10,11,12,13,14,15,16]. Societies that consume a larger amount of omega-3 PUFAs have a lower prevalence of MDD [17]. In addition, patients with MDD have lower omega-3 PUFA levels than healthy controls [18,19]. More importantly, several independent clinical trials and meta-analyses [20,21,22,23,24,25], if not all [26,27,28], have shown that omega-3 PUFAs have antidepressant effects.

Despite the fact that DHA is the main omega-3 PUFA in the brain, EPA is consistently reported to be the most active component of omega-3 PUFAs’ antidepressant effects [16,20,21,22,23,24]. The anti-inflammatory effect is proposed to be associated with the antidepressant effect of EPA, and our recent study further supports this notion by showing that EPA, but not DHA, significantly decreased the incidence of interferon-α-induced depression in HCV patients [15]. In addition, EPA differentiates from DHA in clinical antidepressant efficacy and in upregulating cPLA2 gene regulations at the molecular genetic levels [29]. The treatment of EPA and EPA+DHA has a higher eicosapentaenoylethanolamide (an endocannabinoids derivative of n-3 PUFAs) level than DHA treatment, and eicosapentaenoylethanolamide level is positively correlated with remission rate of MDD [30]. These evidences indicated that nutraceuticals-based therapy may modulate gene expression and induced anti-inflammatory effect, which were associated with personalized medicine toward depression treatments. However, the possible brain circuitry mechanisms of EPA and DHA treatments remained unexplored.

In present study, we investigated the possible central mechanisms of EPA and DHA treatment on MDD patients. More specifically, we explore the possible changes of affective stimuli-evoked brain responses with EPA or DHA intervention in MDD patients. Previous meta-analysis of functional imaging studies reported that the MDD patients revealed abnormal resting-state activities in prefrontal cortex (PFC), temporal cortex, insula, anterior and posterior cingulate cortex, hippocampus, amygdala, and thalamus, whereas the antidepressant treatment may reverse the altered brain activations in these regions [31]. Hence, we hypothesized that the abnormal brain responses to affective stimuli on MDD may be reversed after EPA or DHA treatment.

## 2. Results

### 2.1. Demographic Data and Clinical Outcomes

In 24 MDD patients, 12 patients received EPA treatment and 12 received DHA treatment. There is no significant difference in age, sex, and education years among EPA, DHA, and healthy control groups. There is also no significant difference between EPA and DHA groups in depression episodes and body mass index. For clinical outcomes, no significant interaction effect between groups and times has been found in quantitative HAM-D score. With an overall qualitative remission rate of 62.5% (15 in 24 MDD patients), significantly higher qualitative remission rate has been revealed in EPA group than in DHA group (Table 1). No serious adverse event has been observed in all subjects.

### 2.2. Blood PUFA Level

Compared to the DHA group, significantly increased EPA percentage has been found in the EPA group after treatment. Both EPA and DHA groups revealed significantly increased DHA percentage but decreased arachidonic acid percentage after the treatment. No significant difference has been found for adrenic acid in both groups among different time points (Table 1).

### 2.3. Abnormal Brain Responses between MDD Patients and Healthy Controls

**Figure 1:**
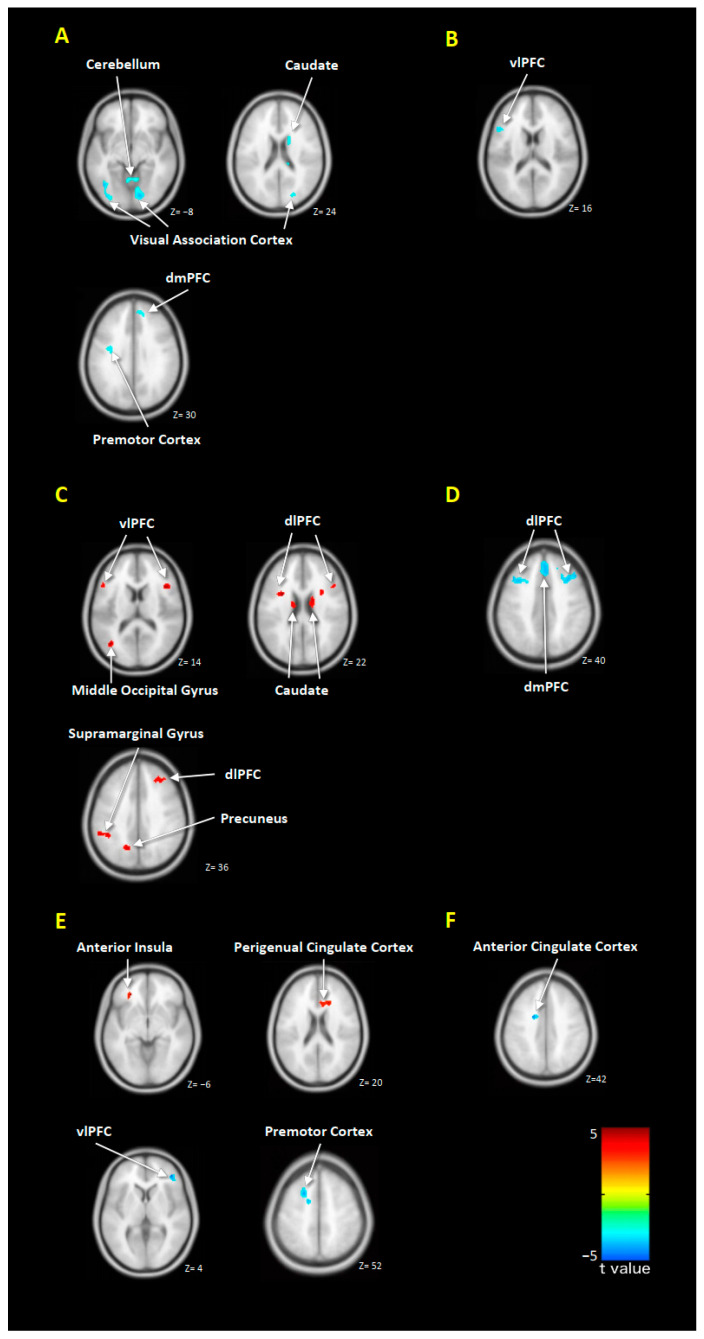
The changes in brain emotional processing in MDD patients. Compared to healthy controls, the untreated MDD patients revealed significant decreased brain responses when processing positive emotion (**A**) and negative emotion (**B**). After treatment, increased brain responses when processing positive emotion has been revealed in EPA group (**C**), while decreased brain responses when processing positive emotion has been revealed in DHA group (**D**). The changes in brain responses were correlated with the changes of HAM-D scores in the EPA (**E**) and DHA (**F**) group. The warm/cold color denotes increased/decreased activity (**A**–**D**) and positive/negative correlation (**E**,**F**), respectively. Abbreviation: dmPFC, dorsomedial prefrontal cortex; dlPFC, dorsolateral prefrontal cortex; vlPFC, ventrolateral prefrontal cortex.

Compared to healthy controls, the untreated MDD patients revealed hypoactivity in the right dorsomedial prefrontal cortex (dmPFC), right caudate nucleus, right lingual gyrus, left premotor cortex, left middle occipital gyrus, and bilateral cerebellum when processing positive emotion (Figure 1A, Supplementary Table S1). When processing negative emotion, untreated MDD patients revealed hypoactivity in the left ventrolateral prefrontal cortex (vlPFC) (Figure 1B, Supplementary Table S1). No more hyperactivity has been found in MDD patients than in healthy controls when processing positive or negative emotion (Supplementary Table S1).

### 2.4. Alterations in Brain Responses after EPA or DHA Treatment

After 12 weeks of EPA treatment, the MDD patients revealed increased brain responses compared to baseline in the bilateral dorsolateral prefrontal cortex (dlPFC), bilateral vlPFC, bilateral caudate nucleus, left middle occipital gyrus, left precuneus, left supramarginal gyrus, and right lingual gyrus when processing positive emotion (Figure 1C, Table 2). No further decreased brain response has been found (Table 2). When processing negative emotion, the MDD patients revealed decreased brain responses than baseline in bilateral dmPFC, left primary somatosensory cortex, left dlPFC, and right caudate nucleus after 12 weeks EPA treatment. No increased brain response has been found (Table 2).

For DHA treatment, the MDD patients revealed decreased brain responses than baseline in bilateral dlPFC and dmPFC when processing positive emotion after 12 weeks treatment. No increased brain response has been found (Figure 1D, Table 2). When processing negative emotion, only the right lingual gyrus revealed increased response compared to baseline after 12 weeks of DHA treatment. No decreased brain response has been found (Table 2).

### 2.5. Correlation between the Changes in Symptoms Severity and Brain Responses

In the EPA group, the change in HAM-D scores and the change in brain responses to positive emotion were positively correlated in left anterior insula and right perigenual cingulate cortex, whereas the negative correlation has been found in the right vlPFC and left premotor cortex (Figure 1E, Table 3). For negative emotions, the change in HAM-D scores and the change in brain responses were positively correlated in the right superior temporal gryus, left inferior parietal lobule, and left posterior cingulate cortex, whereas the no negative correlation has been found in the brain (Table 3).

In the DHA group, the change in HAM-D scores and the change in brain responses to positive emotion were negatively correlated in the left anterior cingulate cortex, whereas no positive correlation has been found in the brain (Figure 1F, Table 3). For negative emotions, the change in HAM-D scores and the change in brain responses were negatively correlated in the left anterior insula, left dmPFC, and left cerebellum, whereas no positive correlation has been found in the brain (Table 3).

## 3. Discussion

To our knowledge, this is the first study to utilize fMRI to investigate the possible central mechanisms of EPA versus DHA treatments in MDD. The main finding is that EPA differentiates DHA treatment by revealing the increased activity in the regions associated with emotion perception and cognitive control when processing the positive emotion. Moreover, the change in HAM-D scores was negatively correlated with the changes in brain response to the positive emotion in the cognitive control regions. In concert with previous studies [21,32], the clinical outcome in the present study also revealed higher treatment efficacy of EPA than DHA. The blood levels of EPA were significantly increased in the EPA group but not in the DHA group, while DHA concentration was significantly increased in both groups. Since higher remission rate and higher blood EPA levels have been observed in the EPA group, these compensatory brain responses may be associated with the central mechanisms of EPA treatment in MDD patients.

The abnormal brain responses to emotion stimuli may be associated with negative affective bias in MDD patients. It has been suggested that dmPFC may be associated with cognitive self-control and impulse regulation [33]. Previous study indicated that lesion of dmPFC may expose the patients to a strong risk of MDD [34]. Almost half of the MDD patients responded to the treatment of excitatory transcranial magnetic stimulation to dmPFC [35]. In addition, the caudate nucleus may associate with motivation-depended responses in healthy subject [36]. In female MDD patients, smaller volumes of caudate nucleus have been found than in healthy controls [37]. Moreover, it has been suggested that the vlPFC may associated with affective response regulation [38,39]. Hence, the hypoactivity in dmPFC and the caudate nucleus when processing the positive emotion, and hypoactivity in vlPFC when processing the negative emotion, may be associated with the impaired affective control of negative affective bias. Besides, it has been long recognized that the lingual gyrus (Brodmann area 18) and middle occipital gyrus (Brodmann area 19) were involved in the visual perception, with increased activity when viewing emotional pictures [40]. The activity in these visual-associated regions may be modulated by cognitive factors such as attention [41]. Thus, the hypoactivity in these visual-associated regions may reflect the impaired affective perception of negative affective bias as the consequences from the interaction between perception and the emotion system.

Despite the anti-inflammatory effect of EPA [15,29,30], the higher treatment efficacy of EPA may be associated with the compensatory and/or resilience brain responses in the regions involved in the cognitive control and emotion perception. After the treatment of EPA, no resilience brain activity has been found in dmPFC but increased brain activity has been found in bilateral dlPFC and vlPFC. Previous meta-analysis on neuroimaging studies suggested that dlPFC plays an important role in the cognitive reappraisal of emotion stimuli [38]. The remission of MDD may accompanied with the activity changes in dlPFC via antidepressant or transcranial magnetic stimulation treatment [42,43]. Besides, it has been suggested that vlPFC is also involved in the responses selection and inhibition within reappraisal processes [44], and interacts with dlPFC during the reappraisal [45]. Thus, the increased activity in bilateral dlPFC and vlPFC after EPA treatment may be associated with the compensatory responses that enhanced cognitive reappraisal processes. These enhanced cognitive reappraisal processes may associate with re-interpreting the meaning of affective stimuli and then change their emotional impacts on MDD patients. This notion has been further supported by the correlation analysis results in present study. The negative correlation has been found between the change of HAM-D scores and the change in brain responses to the positive emotion in vlPFC. Since more decreased HAM-D scores indicated more improvement of clinical symptoms, the higher brain response in vlPFC was associated with more improvement of symptoms of MDD. Moreover, previous study reported that the reappraisal process also enhanced the responses in supramarginal gyrus, precuneus and lingual gryus where they may relate to visual attention [46]. Combined with the resilience activity in the caudate nucleus, the treatment efficacy of EPA may be underpinned by the enhanced reappraisal process to compensate the impaired affective control and emotion perception of negative affective bias.

The lower treatment efficacy of DHA may be associated with no compensatory or resilience response in the brain. After DHA treatment, MDD patients revealed no increased brain response but further decreased brain response in dmPFC and dlPFC when processing positive emotion. Since the dmPFC and dlPFC play an important role in cognitive self-regulation and reappraisal of emotional stimuli [33,38], the decreased brain response in dmPFC and dlPFC may indicate a progressed abnormality to positive emotion stimuli in the DHA group. On the other hand, decreased brain responses in dmPFC, dlPFC, and the caudate nucleus also have been observed in the EPA group when processing negative emotion. These hypoactivities may also indicate a progressed abnormality to negative emotion stimuli in the EPA group. In contrast with the EPA group, no further decreased brain response has been observed in DHA group when processing negative emotion. These findings raised a plausible prevention role of DHA treatment by intercepting the progressed abnormality to negative emotion stimuli. This notion was further supported by the negative correlation between the change in HAM-D scores and the change in brain responses to the negative emotion in dmPFC in the DHA group. The higher brain responses in dmPFC were associated with more improvement of symptoms of MDD. Hence, the interaction between the effect of EPA and DHA in the brain may also play a role to underpin the treatment effect of PUFA in MDD patients.

The supplement form of PUFA may influence brain concentrations on EPA and DHA. A recent animal study suggested that dietary free DHA treatment increased DHA concentrations in blood but not in the brain [47]. Another animal study reported that dietary free EPA treatment increased EPA and DHA levels in blood but only increased EPA level in the brain [48]. On the other hand, treatment of esterified DHA, which can be actively transported across the blood‒brain barrier into the brain [49], increased DHA level in the brain [47]. Similarly, treatment of esterified EPA more increased EPA and DHA level in the brain [48]. Thus, the esterification rate of free EPA and free DHA in peripheral tissue might be another key issue for personalized medicine for MDD. More studies are needed to clarify the possible brain mechanisms of PUFA treatment on MDD.

Several limitations should be noted in the present study. First, a relatively small sample size would lower the statistical power. For clinical outcome (HAM-D score), 22 subjects per group were needed to meet the statistic criteria with alpha = 0.05 and statistical power = 0.8. Hence, to balance the chance of type I and type II error, we applied a less stringent significant threshold on fMRI results. Second, the items of HAM-D were designed for various clinical symptoms of MDD, including mood, insomnia and somatic symptoms. These symptoms may not fully be associated with the negative affective bias. Moreover, one should keep in mind that significant correlation doesn’t imply the causation. Third, we lacked the data of personal history of MDD patients, such as previous medication, hospitalization or refractory symptoms. Fourth, the fMRI has been used to measure the regional neuroactivity changes. The relationship between biochemical and molecular alterations and neuroactivity has not been investigated. Further biochemical or molecular studies may be needed to probe the possible underlining mechanisms to the compensatory but not resilience brain responses of EPA in MDD patients.

In conclusion, our results demonstrated that EPA treatment have more treatment efficacy than DHA treatment in MDD. The hypoactivity in brain regions involved in emotion perception and cognitive control regions may associated with the pathophysiology of MDD. The compensatory brain responses in cognitive reappraisal of emotion and cognitive control regions may underpin the treatment efficacy of EPA. Further large-scale studies may be needed to verify the central mechanisms of different PUFA treatments and to investigate the underlying biochemical and molecular mechanisms of anti-inflammatory nutraceuticals that associated with personalized medicine toward depression treatments.

## 4. Materials and Methods

### 4.1. Subjects

All MDD subjects were recruited from the outpatient clinics of psychiatric department at the China Medical University Hospital, Taichung, Taiwan. The inclusion criteria were as follows: (i) meeting the diagnostic criteria for major depressive disorder in the 4th edition of diagnostic and statistical manual of mental disorders (DSM-IV); (ii) an age of 18 to 65 years old; (iii) a pre-study rating score on the 21-item Hamilton Rating Scale for Depression (HAM-D) [50] of 18 or greater; (iv) being physically healthy from medical history and upon physical examination; and (v) not to have received any psychiatric treatment within 2 weeks. The exclusion criteria for MDD subjects were as follows: (i) a recent or past history of other axis-I diagnoses in DSM-IV besides unipolar major depression, including psychotic disorders, organic mental disorders, impulse control disorders, substance use disorder or substance abuse (in the last six months prior to the studies), and bipolar disorders; (ii) axis-II diagnoses in DSM-IV, i.e., borderline and antisocial personality disorder; (iii) a notable medical comorbidity; (iv) acutely suicidal ideation and attempts such that close monitoring such as hospitalization is necessary; and (v) regular consumption of omega-3 PUFA supplements or a habit of eating fish equal or more than 4 times per week. Every MDD subject was assessed by trained psychiatrists for any psychiatric disorders as determined by the Taiwanese version of structured Mini-International Neuropsychiatric Interview (MINI) [51]. The information for translation, validation, and instruction of the Taiwanese version of MINI can be accessed at the website of Taiwanese Society of Psychiatry (www.sop.org.tw/dow_a.htm).

The research was conducted ethically in accordance with the World Medical Association Declaration of Helsinki. The Institutional Review Board of China Medical University Hospital approved the study. All eligible subjects were received a full explanation of the study and signed written informed consent. The study was registered at Clinicaltrial.gov (N-3 Fatty Acids as the First-line Antidepressant Therapy: From Biomarkers to Clinical Subtypes; Identifier: NCT03871088; https://clinicaltrials.gov/ct2/show/NCT03871088).

### 4.2. Study Design

Twenty-seven eligible MDD subjects participated in this 12-week, double-blind, randomized-controlled trial which commenced in July 2015 and ended in June 2018 when the trial completed. The first subject was recruited on 16 November 2015. For random allocation of the MDD subjects into different treatment groups with a 1:1 ratio, a computer-generated list of random numbers was used in the double-blind randomization procedures. The identical capsules were pre-packed in a plastic zip-bag and consecutively numbered according to the randomization list by an independent nutritionist. The MDD subjects took identical capsules without any information about the PUFA type contained in the capsule. For safety monitoring, the clinical assessments were performed across the whole treatment session. To measure the concentration of PUFA and brain responses to affective stimuli, blood samples were obtained from venous between 0830 and 0930 after overnight fasting and functional magnetic resonance imaging (fMRI) was conducted at baseline (weeks 0) and after treatment (week 12), respectively.

Among these MDD subjects, 1 subject from the EPA group and 2 subjects from DHA group were discontinued to attend the study. Therefore, 24 MDD subjects who completed this 12-week trial were included in the analysis. In order to reveal the possible altered brain responses to affective stimuli in MDD patients, 18 age-matched controls from eligible volunteers were enrolled after exclusion of any psychiatric disorders as determined by the Taiwanese version of MINI (Supplementary Figure S1). Sociodemographic factors, in terms of gender, age, education, past psychiatric history, substances use history, and family history, have been recorded.

### 4.3. PUFA Administration

All MDD subjects took 5 identical capsules per day for 12 weeks. The experimental capsules were weighted 1000 mg and deodorized with orange flavour. Each capsule solely contained concentrated EPA (700 mg) or DHA (350 mg) as the main component, with tertiary-butyl hydroquinone (0.2 mg) and tocopherols (2 mg) as antioxidants. The sources of EPA and DHA were from anchovy fish body oil (AK BioTech, Ulsan, Korea) and algal vegetable (DSM Nutritional Products, Basel, Switzerland), respectively.

### 4.4. Measurement for Blood Pufa Level

The measurement of blood PUFA level were conducted on coded blood samples by the staff who were blinded to information about the subjects. The blood samples were prepared within 24 h to separate erythrocyte for analysis of variant fatty acids by gas chromatography of methyl esters (Lipid Standards, FAME, Sigma Co., St. Louis, MO, USA). The detailed step-by-step procedures have been published elsewhere [52]. Fatty acid profiles were identified by comparing the retention times with those of appropriate standard fatty acid methyl esters. The levels of each fatty acid were expressed as a percentage of total fatty acids.

### 4.5. Clinical Assessments

The judgment of concomitant medications for anxiety and insomnia or discontinuation from this trial for re-allocation to antidepressant drugs was based on physicians’ clinical judgment. The 21-item HAM-D was used to assess the depressive symptoms as clinical outcomes before (week 0), during (week 1, 2, 4, 8), and after treatment session (week 12). The remission of clinical symptoms was considered if the HAM-D score was less than 7.

### 4.6. Stimulation Paradigm of FMRI

The images used for affective stimulation were selected from the international affective picture system (https://csea.phhp.ufl.edu/index.html). To maximize the effect of emotional stimuli, pictures of positive emotion with high arousal level, negative emotion with high arousal level, and neutral emotion were used. Equal numbers of positive, negative, and neutral pictures were presented to each subject within 4 fMRI sessions. In each session, pictures of different emotional conditions were presented in 3 blocks with a counterbalanced order across subjects. Each block presented one emotional condition (4 pictures, 6 s per picture) and interleaved with a 24-s fix-cross block as baseline control (Supplementary Figure S2). Pictures and fix-cross were projected onto a screen with a rear screen projector. Subjects perceived stimuli with a refectory mirror attached on the head coil.

### 4.7. Image Acquisition

Images were acquired with an 8-channel head coil in a 3.0 Tesla GE MRI scanner (Signa HDxt, GE Healthcare, Chicago, IL, USA). The fMRI sessions were continuously scanned with ascending interleaved echo-planar imaging sequence for whole brain scanning (repetition time = 2000 ms; echo time = 30 ms; flip angle = 90°; matrix = 64 × 64; field of view = 240 × 240 mm^2^; slice number = 32; slice thickness = 4.4 mm). Prior to the first block of emotional stimulation in each fMRI session, 10 s blank scans were conducted to stabilized the signal. High-resolution, 3-dimensions T1-weighted anatomical images were also acquired with spoiled gradient echo sequence (repetition time: 7.344 ms; echo time: 2.728 ms; flip angle = 12°; matrix = 256 × 256 × 170; field of view = 224 × 224 × 170 mm^3^). All scans were acquired within a dim-light shielding room. Before scanning, subjects were instructed to stay relaxed but not to move the head, and to fixate their attention on the stimulation presented in the refractory mirror.

### 4.8. Preprocessing of fMRI Data

Before the data preprocessing, the blank scans were discarded from each fMRI session. The remaining fMRI images were then preprocessed by statistical parametric mapping 12 (SPM12, Wellcome center for Human Neuroimaging, University College London, London, UK). Within each session, the serial fMRI images were firstly corrected for different slice acquisition times and then realigned to correct the head motions occurring alone the session. Subjects who had more than 3 mm translation or 3° of angular rotation were excluded from further analysis. The T1-weighted anatomical image was then coregistrated to the mean image which was generated from the previous realignment step. After the coregistration step, the anatomical image was normalized to the default template of tissue probability map in SPM 12. The normalization parameters were then applied to all fMRI images within the session to normalize them into Montreal Neurological Institute (MNI) reference space and resampled with the voxel size 2 × 2 × 2 mm^3^. Finally, the normalized fMRI images were smoothed with a 3-dimensional Gaussian kernel (8 mm full-width at half-maximum).

### 4.9. Statistics

The statistical analysis for demographic data, clinical outcome, and blood PUFA levels were conducted using SPSS 21 (IBM Inc., Armonk, NY, USA). To test the possible difference among EPA, DHA, and healthy control groups, one-way analysis of variance (ANOVA) and Chi-square were conducted on continuous data and categorical data, respectively. The mixed-model two-way ANOVA and Chi-square were conducted to test the possible difference between groups (EPA and DHA) and times (week 0 and 12) on continuous data and categorical data, respectively. The significance was considered if *p* < 0.05.

For fMRI data, the conventional 2-level (individual and group level) analysis has been conducted using generalized linear model in SPM 12. At the first level, individual brain responses for each emotion condition were modelled through the stimulation paradigm which convoluted with a canonical hemodynamic response function. A high-pass filter of 1/128 Hz and an AR (1) model were used to remove the low frequency drafts and correct for temporal auto correlations. Scans from all 8 sessions (4 from the baseline scans and 4 from the after-treatment scans) were included in the model. Six head motion parameters obtained from the realignment step were further used as covariates in the model. The maps of estimated beta value for each emotion condition were calculated and then the following relevant maps of beta contrasts were computed for second level analysis. The positive and negative emotion stimulations were contrasted against the neutral stimulations to reveal the associated brain regions for positive and negative emotion processing, respectively. The maps of positive and negative emotion processing between different time point (before and after treatment) were also computed to reveal the possible changes of brain responses for each emotion condition, respectively.

In second level analysis, several pre-planned tests have been conducted to probe the possible difference between groups and times. For disease-related abnormality, 2-sample t-tests were conducted between all untreated MDD patients (i.e., data acquired before treatment) and healthy subjects on positive and negative emotion conditions, respectively. For treatment-related effect, 1-sample t-tests were conducted on positive and negative emotion conditions between different time points of EPA or DHA treatment, respectively. The correlation analysis was also conducted between the change of clinical symptoms severity (HAM-D scores) and the change of brain responses to positive and negative emotion, respectively. Considering that the fMRI study often have low statistical power [53], a less stringent significant threshold (uncorrected *p* < 0.005 at voxel-level with the cluster size > 50, corresponding to the uncorrected cluster-level *p* < 0.34) was applied in the present study to balance the chance of Type I and Type II error as suggested by a previous study [53].

## Figures and Tables

**Table 1:** Demographic data, clinical measurements, and concentration of blood polyunsaturated fatty acids.

	EPA	DHA	Control	*p* Value
	(N = 12)	(N = 12)	(N = 18)	
Sex	3 M/9 F	4 M/8 F	7 M/11 F	0.732
Age (year-old)	45.42 ± 9.756	42.50 ± 12.573	45.22 ± 8.063	0.715
Education (year)	12.58 ± 4.188	15.00 ± 2.486	13.11 ± 2.166	0.116
BMI	23.17 ± 3.46	23.52 ± 4.03		0.824
Episodes				0.237
1	7	5		
2	3	2		
3	0	4		
4	1	1		
5	1	0		
HAM-D				0.101
Week 0	25.42 ± 3.579	25.75 ± 4.309	-	
Week 12	4.83 ± 2.691	9.33 ± 6.88	-	
Remission				0.035 *
Yes	10	5	-	
No	2	7	-	
PUFA level (%)				
EPA			-	0.006 *
Week 0	2.55 ± 0.95	2.60 ± 0.90	-	
Week 12	4.70 ± 1.27	2.87 ± 1.03	-	
DHA				0.42
Week 0	4.04 ± 0.68	4.17 ± 1.11	-	
Week 12	4.95 ± 1.35	5.49 ± 1.74	-	
AA				0.106
Week 0	6.24 ± 1.45	6.27 ± 1.76	-	
Week 12	3.67 ± 1.57	4.85 ± 1.27	-	

BMI: body mass index; PUFA: polyunsaturated fatty acids; EPA: eicosapentaenoic acid; DHA: docosahexaenoic acid; AA: arachidonic acid; HAM-D: Hamilton Depression Rating Scale; M: male; F: female. *: *p* < 0.05. Most data expressed in mean ± SD except “Sex” and “Remission” expressed in number.

**Table 2:** The changes in brain response to different emotion stimuli after eicosapentaenoic acid or docosahexaenoic acid treatment.

Anatomic Area	BA	Size	t Score	Coordinates (mm)		
				x	y	z
EPA treatment						
Positive Emotion						
Increased activity						
L Mid Occipital G	18	760	6.31	−22	−96	2
R Lingual G	17	278	5.84	18	−92	−8
B Sup Frontal G	9	51	4.70	−30	8	22
	9	168	4.68	30	22	28
B Inf Frontal G	45	98	4.51	44	20	14
	45	64	4.45	−52	22	10
B Caudate N		91	4.43	−14	−10	24
		66	4.16	16	−2	22
L Precuneus	7	67	4.16	−14	−64	36
L Supramarginal G	40	80	3.99	−44	−44	36
Decreased activity						
NS						
Negative Emotion						
Increased activity						
NS						
Decreased activity						
B Med Frontal G	9	572	5.45	−4	46	26
	8		5.03	2	28	54
L Postcentral G	2	314	4.89	−44	−30	46
R Caudate N		301	4.73	10	12	18
L Mid Frontal G	9	82	3.83	−40	6	42
DHA treatment						
Positive Emotion						
Increased activity						
NS						
Decreased activity						
B Med Frontal G	8	1231	5.66	−6	28	52
			5.49	4	30	46
R Sup Frontal G	8		4.30	30	18	56
L Sup Frontal G	8	207	4.80	−28	18	56
Negative Emotion						
Increased activity						
R Lingual G	18	58	3.92	14	−80	−6
Decreased activity						
NS						

BA: Brodmann area; Size: number of voxels in the cluster; EPA: eicosapentaenoic acid; DHA: docosahexaenoic acid; L: left; R: right; B: bilateral; Sup: superior; Inf: inferior; Mid: middle; Med: medial; G: gyrus; N: nucleus; NS: no significant cluster.

**Table 3:** Correlation between the changes of brain responses to emotion stimuli and the changes of Hamilton depression rating scale.

Anatomic Area	BA	Size	t Score	Coordinate (mm)		
				x	y	z
EPA treatment						
Positive Emotion						
Positive correlation						
L Ant Insula	11	68	5.34	−26	38	−6
R Perigenual Cingulate G	32	71	4.61	18	26	20
Negative correlation						
R Inf Frontal G	10	88	5.42	42	44	4
L Sup Frontal G	6	123	4.67	−20	12	52
Negative Emotion						
Positive correlation						
R Sup Temporal G	22	82	4.51	48	−36	6
L Inf Parietal Lobule	40	70	4.11	−48	−48	42
L Post Cingulate Cortex	29	52	3.81	−6	−42	20
Negative correlation						
NS						
DHA treatment						
Positive Emotion						
Positive correlation						
NS						
Negative correlation						
L Ant Cingulate G	24	61	5.17	−18	2	42
Negative Emotion						
Positive correlation						
NS						
Negative correlation						
L Ant Insula		60	7.97	−30	10	12
L Med Frontal G	6	236	5.76	−16	−6	50
L Cerebellum		64	4.04	−18	−58	−8

BA: Brodmann area; Size: number of voxels in the cluster; EPA: eicosapentaenoic acid; DHA: docosahexaenoic acid; L: left; R: right; Ant: anterior; Post: posterior; Sup: superior; Med: medial; Inf: inferior; G: gyrus; NS: No Significant Cluster.

## Supplementary Materials

The following are available online at https://www.mdpi.com/2075-4426/10/4/283/s1, Supplementary Figure S1: The CONSORT flowchart of the study. Supplementary Figure S2: Stimulation paradigm for functional magnetic resonance imaging. Supplementary Table S1: Abnormal hypo-responses to different emotion stimuli on untreated major depressive disorder.
