# Supplementary material for: Brain Responses to Emotional Stimuli after Eicosapentaenoic Acid and Docosahexaenoic Acid Treatments in Major Depressive Disorder: Toward Personalized Medicine with Anti-Inflammatory Nutraceuticals
Source: J Pers Med. 2020 Dec 16;10(4):283. doi: 10.3390/jpm10040283 (PMC7765544; doi:10.3390/jpm10040283)
Supplement: Supplementary file 1 [file jpm-10-00283-s001.pdf]

Content in the current online supplementary materials

Figure S1. The CONSORT flowchart of the study. 2
Figure S2. Stimulation paradigm for functional magnetic resonance imaging...... 3

Table S1. Abnormal hypo-responses to different emotion stimuli on untreated major depressive disorder.... 4

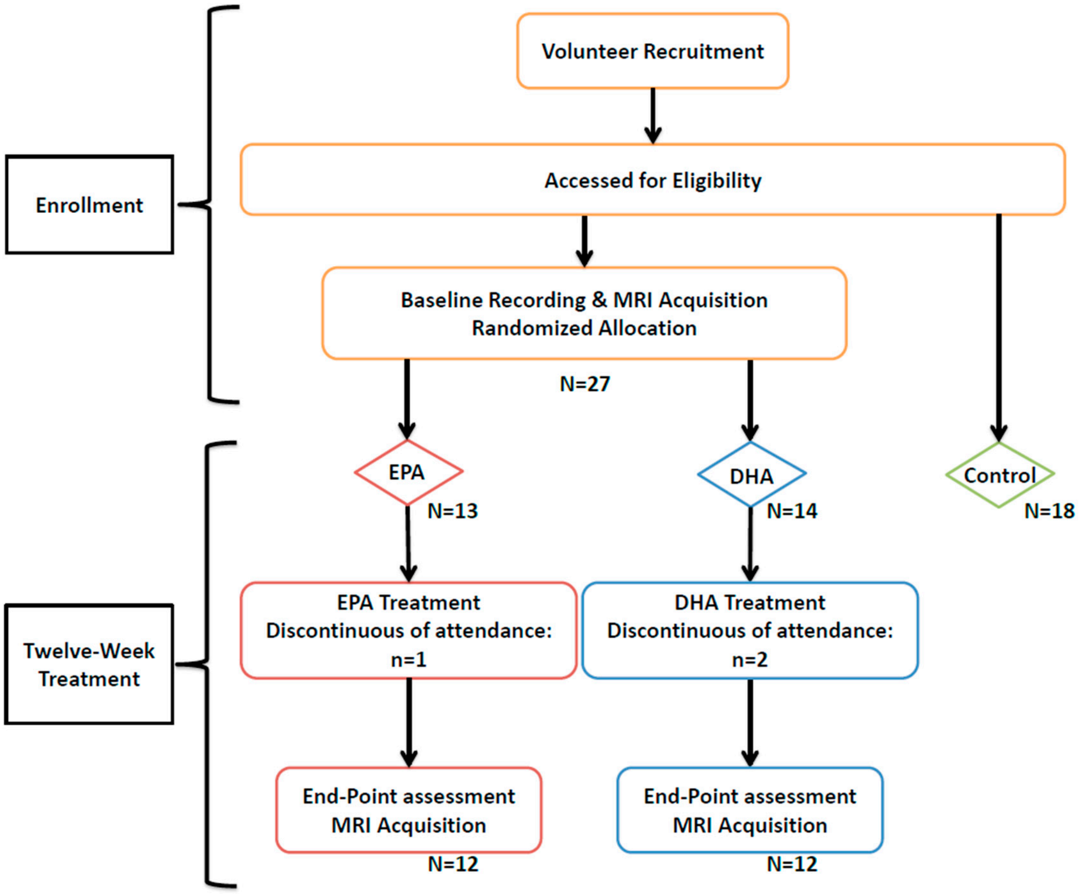

Figure S1. The CONSORT flowchart of the study.

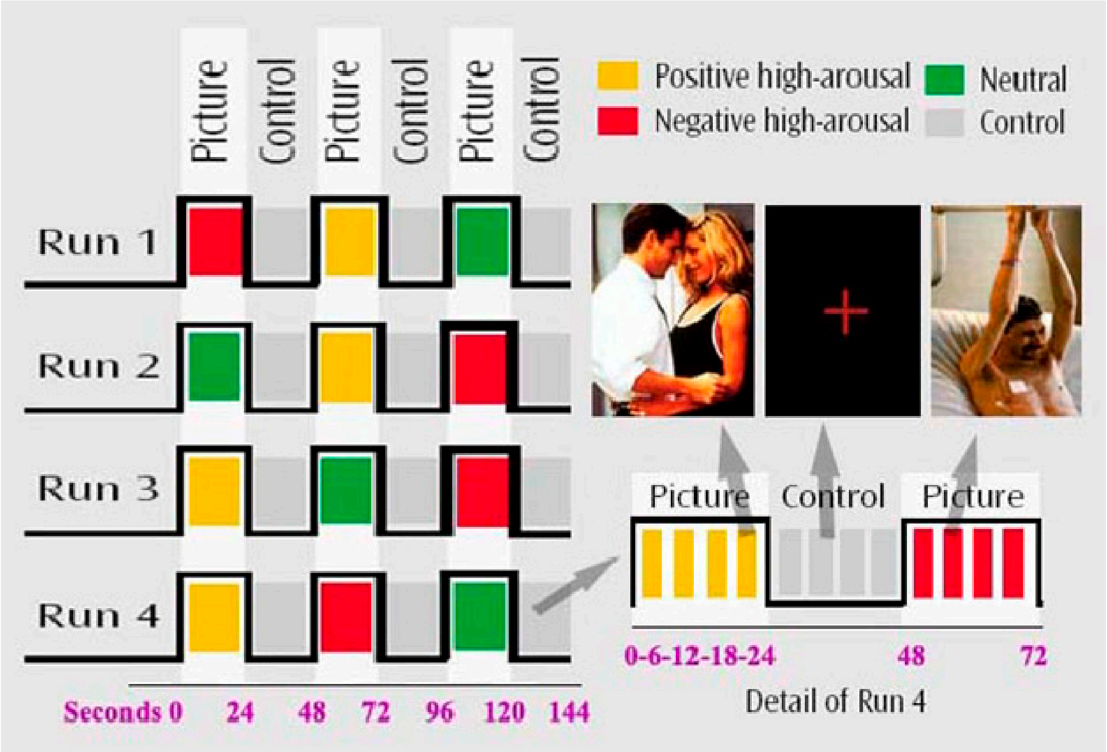

Figure S2. Stimulation paradigm for functional magnetic resonance imaging. Before and after the treatment session, 4 functional magnetic resonance imaging (fMRI) sessions have been conducted to measure the brain responses evoked by emotion pictures. In each fMRI session, pictures of positive, negative, and neutral emotion were presented in separate 3 blocks with a counterbalanced order across subjects. Each block contained 4 pictures of single emotion for total 24 seconds. Between blocks, a 24-seconds fix-cross was presented served as baseline control.

Table S1. Abnormal hypo-responses to different emotion stimuli on untreated major depressive disorder.

Anatomic area	BA	Size	t Score	Coordinates (mm)		
				x	y	z
Positive Emotion						
L Mid Occipital G	19	726	4.35	-40	-66	-2
R Lingual	18	516	3.76	16	-80	-10
L Precentral G	6	67	3.47	-34	-8	32
R Med Frontal G	9	50	3.43	10	48	28
R Caudate N		72	3.42	16	2	24
B Cerebellum		84	3.11	-4	-56	-8
			2.96	6	-56	-8
Negative Emotion						
L Inf Frontal G	45	67	3.52	-54	20	14

BA: Brodmann area; Size: number of voxels in the cluster; L: left; R: right; B: bilateral; Mid: middle; Inf: inferior; Med: medial; G: gyrus; N: nucleus.
